# Continuity of care in the approach to cardiac patients: from theory to practice/Continuidad asistencial en el abordaje del paciente cardiaco: de la teoría a la práctica

**Published:** 2012-05-29

**Authors:** Pilar Sánchez Chamero, Luis Miguel Ceresuela, Eric Sitjas Molina, Olga Guri Bairet, Elena Casanova, Jordi Juliá Gibergans

**Affiliations:** Integrated Health Consortium (CSI, Consorci Sanitari Integral), Hospitalet General Hospital (HGH), L’Hospitalet de Llobregat, Spain; CSI, HGH, L’Hospitalet de Llobregat, Spain; CSI, HGH, L’Hospitalet de Llobregat, Spain; CSI, HGH, L’Hospitalet de Llobregat, Spain; CSI, HGH, L’Hospitalet de Llobregat, Spain; CSI, HGH, L’Hospitalet de Llobregat, Spain

**Keywords:** continuity of care, specialised care, primary care, continuidad asistencial, atención especializada, atención primaria

## Introduction

The Department of Cardiology of our hospital has a catchment population of 135,000 people. Until 2005, the relationship between the various levels of care (primary care, and secondary and tertiary hospitals) was incidental. Patients were seen at different levels of care and complementary tests were duplicated due to the lack of connections between these levels, with all the implications this has for patients, quite apart from the associated costs for the health service.

In 2005, the CSI HGH took charge of cardiology care in the catchment area, integrating the outpatient cardiology care of five primary care (PC) health districts (Florida Norte, Florida Sur, Pubilla Cases, Collblanc and Torrasa). In the first year, 12,000 appointments were carried out. The first intervention put into place was to transfer the care of all the monitored patients with no identified heart disease or with only minor conditions to PC. With this step, we managed to reduce the number of visits to specialists of the Department of Cardiology by 30% in two years. Another intervention was introduced in 2007: the establishment of clinical sessions bringing together professionals of the Department of Cardiology and PC. A cardiologist was appointed for referral of non-complex chronic heart patients and for advising primary care colleagues. Despite all this, the response was not consistent across PC, so we proposed to segment the cardiology care offer, according to the needs of our internal client, that is, primary care.

## Description of the intervention

The objective was to redefine cardiac care processes towards patient-based care through a model of care that provides specialised care tailored to the needs of the ‘client’ within the care system as well as of patients. The intervention involved:

- Establishment of an integrated care plan (ICP) to guide the approach to patients with heart failure:
  - Establishment of a single appointment for diagnosis of heart failure (HF), including tests and preparation of the care plan
  - Standardise response times for carrying out complementary tests
  - Introduce congestive heart failure (CHF) nurse case managers
  - Establish mechanisms for communication with PC that compensate for the lack of shared medical records
  - Agree with PC the criteria for patient referral, as well which area of the hospital patients should be sent to
  - Agree on the essential data required across the various levels of care
  - Establish a set of indicators for monitoring the criteria for referral, the volume of activity in the various areas, and the time patients take to progress along the care pathway
- Setting up of a high-resolution cardiac imaging unit (CIU), establishing an agreed number of processes and standard time (1 week) for performing of tests at the hospital level.
  - Agree with PC on the types of cases that could benefit from a visit to this unit (appointment with the nurse manager, electrocardiogram, echocardiogram, ± plain radiography)
- Setting up of a service of virtual consultations for minor conditions and queries from PC professionals, for cases in which a visit to the cardiologist is not required.
- Define the activities and duties of the professionals of each level of care in relation to each care process.

## Results

1. The various interventions in place up to 2010 led to a 50% reduction in annual outpatient consultations (2365 first appointments, 4538 follow-ups) and increased the capacity of PC to deal with cardiac conditions, achieving a follow-up to first appointment ratio of 1.92 in cardiology and waiting times for specialists of less than a week.

2. Outcomes of the ICP and referrals for rapid diagnosis of CHF from March 2010 to July 2011: Overall, 112 patients ranging from 43 to 90 years of age have been assessed (Table 1).

**Figure 1.**
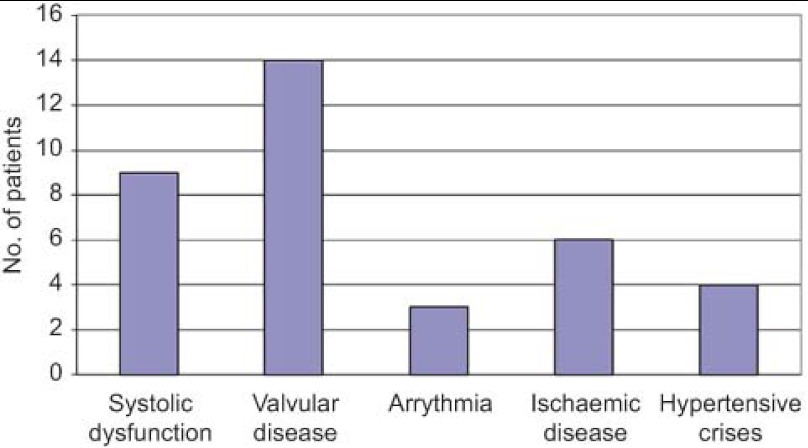
HF confirmed in 36 patients.

Of the patients examined, HF was confirmed in 36 cases and the underlying causes are detailed in Figure 1.

3. Outcomes of the high-resolution CIU from June to July 2011: A total of 20 patients were assessed. Patient age ranged from 25 to 88 years old, with 9 patients being octogenarians. The reasons for the referral are listed in Table 2.

**Figure 2.**
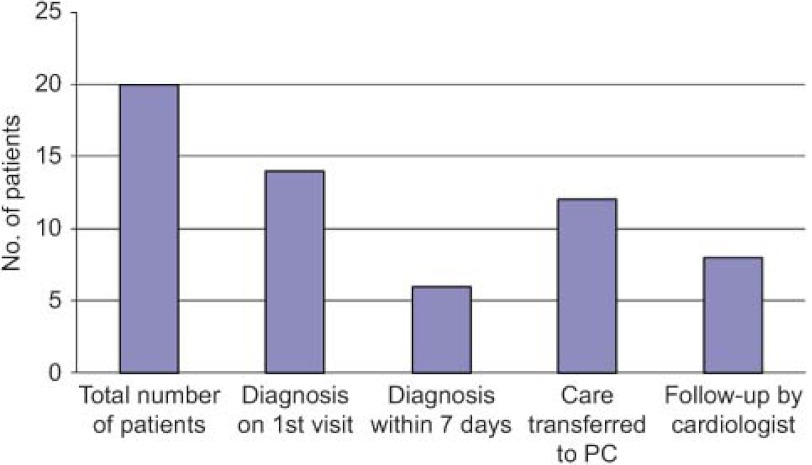
Achievement of diagnosis and type of referral.

The outcomes with respect to the achievement of diagnosis for the processes that had been the reason for the consultation are reported in Figure 2.

4. Outcomes of the virtual consultations: Over a period of three months, 34 consultations were carried out and, of these, 32 have were resolved virtually. It was only considered necessary for two patients to attend appointments in person to be assessed (in the high-resolution CIU or at a conventional specialist appointment).

## Discussion

Until 2005, the relationship between levels of care was incidental. Despite the fact that there were significant initiatives between primary and specialised care, well received by the latter, a direct connection had not developed between levels of care. Since the time cardiology care came under the responsibility of our hospital, a series of interventions have been put in place in order to agree with primary care, as well as within tertiary care, pathways and criteria for action in line with clinical practice guidelines to provide continuity of care to patients regardless of the level of care where they are treated.

## Conclusions

Consensus among the various different levels of care involved in the process, from a client-provider (PC-Hospital) perspective, is essential to determine, on the basis of care needs and the principle of subsidiarity, who does what and where.

With the establishment of new practices in integrated patient care, we aim to respond to the care processes that are the reason for consultations quickly and efficiently.

Our vision is to develop a cardiology unit capable of adapting its responses to care process requirements as requested by its internal client.

## Conference abstract Spanish

## Introducción

El servicio de cardiología de nuestro hospital da cobertura a una población total de 135.000 habitantes. Hasta el año 2005 la relación entre los diferentes niveles asistenciales (primaria, hospitales de 2° y 3er nivel) eran circunstanciales. El paciente acudía a los diferentes niveles y se producía una duplicidad de exploraciones complementarias, como consecuencia de la falta de conexión, con las implicaciones que conlleva para el paciente y el gasto sanitario.

En el año 2005 el C.S.I-HGH se hace cargo de la Atención Cardiológica (AC) del área de referencia, integrando la AC ambulatoria de cinco áreas básicas de Atención Primaria (AP) (Florida Norte, Florida Sur, Pubilla Cases, Collblanc y Torrasa). En el primer año se realizaron 12.000 visitas. La primera intervención que se realizó fue remitir a AP el 100% de los pacientes controlados que no tenían patología cardiaca o con patología banal. Con esta intervención se consigue en 2 años, reducir un 30% las visitas de cardiología. A partir del 2007 se realiza una segunda intervención, que es la creación de sesiones conjuntas entre el servicio de cardiología y la AP. Se crea el cardiólogo referente para poder derivar a los pacientes cardiópatas crónicos no complejos y la orientación al cliente interno. A pesar de todo, la respuesta por parte de la AP no es homogénea, por lo que nos planteamos segmentar adecuadamente nuestra oferta asistencial cardiológica a las necesidades de nuestro cliente interno Atención Primaria.

## Descripción de la intervención

El objetivo de la intervención es redefinir los procesos asistenciales cardiológicos, poniendo en el eje central al paciente a través de un modelo asistencial que dé respuesta especializada a la necesidad del nivel asistencial ‘cliente’ y del paciente.

La intervención supuso (2010):

- Puesta en marcha de un Plan Asistencial Integrado (PAI) para el abordaje de los pacientes con Insuficiencia Cardiaca: Creación de un dispositivo de visita única para el diagnóstico de Insuficiencia Cardiaca (IC)
  - Estandarizar tiempos de respuesta para la realización de pruebas complementarias
  - Implementar enfermera gestora de la insuficiencia cardiaca congestiva (ICC)
  - Instaurar sistemas de comunicación con la AP, que compensen la falta de una historia clínica unificada.
  - Acordar con la AP criterios de derivación de pacientes y el dispositivo hospitalario al cual remitir al paciente.
  - Acordar la información imprescindible entre los diferentes niveles asistenciales.
  - Implantar un sistema de indicadores para el seguimiento de los criterios de derivación, el volumen de actividades de las diferentes áreas, y el tiempo de ciclo del proceso
- Puesta en marcha de una Unidad de Alta Resolución (UAR) para patología cardíaca, establecimiento de un número de procesos acordados y estandarización de los tiempos (1 semana) para las exploraciones a nivel hospitalario.
  - Definir de forma consensuada con AP los procesos que se pueden beneficiar de una visita de alta resolución (visita enfermera gestora, electrocardiograma, ecocardiograma, ± radiología simple)
- Puesta en marcha de un servicio de consultaría no presencial para patología banal y dudas por parte de la AP que no requiera visita presencial por parte del cardiólogo.
- Definir las actividades y responsabilidades de cada nivel asistencial en base a cada proceso.

## Resultados

1. Las diferentes intervenciones hasta el 2010, han permitido disminuir la actividad ambulatoria anual inicial en un 50% (2.365 primeras visitas, 4.538 segundas visitas), incrementar la capacidad resolutiva de la AP en patología cardíaca, conseguir una tasa de reiteración en las consultas de cardiología de 1,92, y ser accesibles en <1 semana.

2. Resultados PAI + derivaciones a diagnóstico rápido ICC en el periodo comprendido entre marzo 2010 y julio 2011: Se han valorado un total de 112 pacientes, con un intervalo de edad de 43–90 años (Tabla 3).

**Figura 3.**
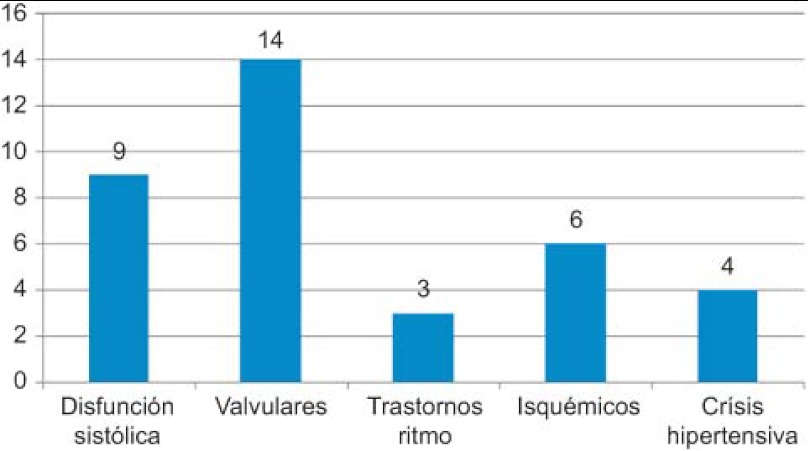
IC confirmada en 36 pacientes.

De los pacientes remitidos se ha confirmado el diagnostico de Insuficiencia cardiaca en 36 pacientes y las causas quedan reflejadas en la Figura 3.

3. Resultados UAR en el periodo de junio y julio 2011: Se han valorado un total de 20 pacientes, con un intervalo de edad de 25 a 88 años, de los cuales 9 pacientes tenían más 80 años. El motivo de la derivación se describe en la Tabla 4.

**Figura 4.**
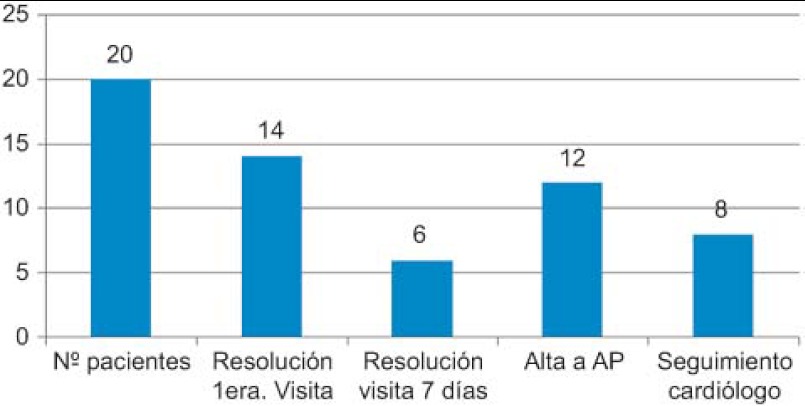
Resolución de proceso diagnóstico y tipo de derivación.

Los resultados de la resolución del proceso que ha motivado la consulta y el tipo de derivación se muestran en la Figura 4.

4. Resultados de la consultoría no presencial: Se han valorado en un periodo de 3 meses un total de 34 consultas que se han solucionado en 32 casos. Sólo en dos de las consultas ha sido necesaria la valoración presencial (por la UAR o en consulta ordinaria).

## Discusión

Hasta el año 2005 en nuestro entorno asistencial la relación entre los diferentes niveles era testimonial. A pesar de que ha habido iniciativas importantes desde la AP- Atención Especializada, con buena acogida por ésta última, no ha llegado a cristalizar una relación directa interniveles. A partir de que la AC es asumida por nuestro hospital, se ponen en marcha toda una serie de intervenciones, con la finalidad de consensuar con la primaria, así como con el tercer nivel asistencial, las rutas y los criterios de actuación según las guías de práctica clínica, para dar continuidad asistencial al paciente independientemente del nivel asistencial donde sea tratado.

## Conclusiones

El consenso entre los diferentes niveles asistenciales implicados en el proceso, desde una visión de cliente proveedor (AP-Hospital), es imprescindible para determinar en base a la necesidad asistencial y el principio de subsidiaridad quien hace que y donde se realiza.

Con la puesta en marcha de las nuevas medidas en la atención integral del paciente, se espera dar respuesta al proceso que motiva la consulta en la mayor parte de los casos de forma breve y eficaz. Nuestra visión es disponer de un servicio de cardiología capaz de adecuar sus respuestas asistenciales a la necesidad asistencial del proceso solicitado desde el nivel asistencial cliente.

## Figures and Tables

**Table 1.** Outcomes of the rapid diagnosis of heart failure

Characteristic	Number of patients
Women	69
>80 years old	23
High blood pressure	70
High blood pressure + type 2 diabetes	34

**Table 2.** Reasons for referral

Reason for referral	Number of patients
Arrhythmia	11
Chest pain	4
Syncope	3
Valvular disease	1

**Tabla 3.** Resultados del diagnóstico rápido de IC

Característica	Número de pacientes
Mujeres	69
>80 años	23
HTA	70
HTA+DMII	34

**Tabla 4.** Motivo consulta (junio-julio 2011)

Motivo consulta	Número de pacientes
Trastornos del ritmo	11
Dolor torácico	4
Sincope	3
Valvulopatías	1
